# Data set from gas sensor array under flow modulation^☆^

**DOI:** 10.1016/j.dib.2015.02.016

**Published:** 2015-03-04

**Authors:** Andrey Ziyatdinov, Jordi Fonollosa, Luis Fernández, Agustín Gutiérrez-Gálvez, Santiago Marco, Alexandre Perera

**Affiliations:** a B2SLab, Department of ESAII, Universitat Politenica de Catalunya, Pau Gargallo 5, Barcelona, Spain; b Centro de Investigacion Biomedica en Red en Bioingenierıa, Biomateriales y Nanomedicina (CIBER-BBN), Barcelona, Spain; c BioCircuits Institute, University of California, San Diego, La Jolla, CA 92093, USA; d Signal and Information Processing for Sensing Systems Institute for Bioengineering of Catalonia (IBEC), Baldiri Reixac, 4-8, 08028 Barcelona, Spain; e Departament d׳Electronica, Universitat de Barcelona, Marti i Franques 1, 08028 Barcelona, Spain

**Keywords:** Gas sensor array, MOX sensor, Flow modulation, Early detection, Biomimetics, Respiration, Sniffing

## Abstract

Recent studies in neuroscience suggest that sniffing, namely sampling odors actively, plays an important role in olfactory system, especially in certain scenarios such as novel odorant detection. While the computational advantages of high frequency sampling have not been yet elucidated, here, in order to motivate further investigation in active sampling strategies, we share the data from an artificial olfactory system made of 16 MOX gas sensors under gas flow modulation. The data were acquired on a custom set up featured by an external mechanical ventilator that emulates the biological respiration cycle. 58 samples were recorded in response to a relatively broad set of 12 gas classes, defined from different binary mixtures of acetone and ethanol in air. The acquired time series show two dominant frequency bands: the low-frequency signal corresponds to a conventional response curve of a sensor in response to a gas pulse, and the high-frequency signal has a clear principal harmonic at the respiration frequency. The data are related to the study in [1], and the data analysis results reported there should be considered as a reference point.

The data presented here have been deposited to the web site of The University of California at Irvine (UCI) Machine Learning Repository (https://archive.ics.uci.edu/ml/datasets/Gas+sensor+array+under+flow+modulation). The code repository for reproducible analysis applied to the data is hosted at the GutHub web site (https://github.com/variani/pulmon).

The data and code can be used upon citation of [1].

**Specificatioins Table**

**Table:** 

Subject area	Chemistry, Biology, Engineering
More specific subject area	Chemometrics, Machine Olfaction, Electronic Nose, Chemical Sensing, Machine Learning
Type of data	Table
How data was acquired	A custom set up based on a PC104-standard embedded computer, an array of commercial metal-oxide gas sensors (TGS 2610, TGS 2602, TGS 2600, TGS 2611, and TGS 2620 models; Japan Figaro Engineering Inc.), and a mechanical ventilator (Inspira Advanced Safety Ventilator; Harvard Apparatus).
Data format	Raw, analyzed
Experimental factors	Dilutions of analytes or binary mixtures of analytes (volume concentration) were prepared in advance. Previously to the measurements, the respiration frequency of the ventilator was empirically evaluated to be 5 breaths per minute, such that the rate of the gas flow was low enough for the temporal dynamics of the sensors. The measurements were collected in a period of 4 days to minimize the effect of the long-term internal and environmental noise in the system.
Experimental features	10 μL of a dilution was delivered to the vessel using a micropipette. The mechanical ventilator took the air sample from the vessel and pushed it to the measurement chamber, simulating respiration cycles with the period of 12 s. The transient signals from the sensors were acquired at a frequency of 25 Hz during 5 min.
Data source location	Barcelona, Spain
Data accessibility	The data set is stored in The University of California at Irvine (UCI) Machine Learning Repository (https://archive.ics.uci.edu/ml/datasets/Gas+sensor+array+under+flow+modulation). The code repository for reproducible analysis applied to the data set is hosted at the GutHub web site (https://github.com/variani/pulmon). Citation of [1] is required.

**Value of the data**

• The data provide insights into the role of active sampling in the olfactory system.
• The findings might be on the focus of the early detection scenario.
• The data suit for different pattern recognition tasks in Machine Olfaction, mainly a multivariate regression with multiple responses.
• The dataset can be used to explore sensor redundancy in artificial gas sensor arrays.

## 1 Experimental design, materials and methods

### 1.1 Experimental set up

The array was composed of 16 metal-oxide gas sensors of 5 different TGS models from Figaro Inc. [2]. The sensors were configured for 10 different sensor conditioning profiles based on the combination of 5 TGS models and 2 sensor operating temperatures. Table 1 reports the configuration parameters of the sensors. The circuit board with the gas sensor array was placed in a 70 ml inner volume chamber connected to the mechanical ventilator.

The device of the mechanical ventilator was made commercially available from Harvard Apparatus [3]. The ventilator includes a cylinder of volume 63.44 cm^3^, a mechanical pump and three outlets, namely, ‘Source’, ‘To Animal’ and ‘From Animal’. The pump takes air from the outlet ‘Source’ and pushes the air sample through the outlet ‘To Animal’. The system also receives the sample again in the outlet ‘From Animal’, such that the loop is closed. The chamber with the sensors is interconnected with both ‘To Animal’ and ‘From Animal’ channels. The resulted gas flow modulation system also controls the air pressure decay and collects the exhaled air. The cylinder of the ventilator was fixed to a frequency of 5 breaths per minute, approximately equivalent to 0.08 Hz.

The acquisition of sensor signals was performed by a PC104-standard embedded computer, which was designed for real-time acquisition, processing and visualization of sensory data for an autonomous mobile robot [4]. The voltage output lines of the circuit board were read by the 16-bit Analog-to-Digital Converter (ADC) board with 16 channels acquiring at a frequency of 25 Hz. The embedded computer ran a custom built GNU/Linux image designed for a refined control of the measurement process.

## 2 Measurement protocol

Three concentrations doses 0.1, 0.3 and 1 vol.% were used to prepare the dilutions in water for the pure analytes. The same analyte concentrations were used to prepare mixtures. The gas classes included samples of pure ethanol (‘lab’ attribute eth-0.1, eth-0.3 and eth-1), samples of pure acetone (ace-0.1, ace-0.3 and ace-1), samples of binary mixtures of ethanol and acetone (ace-0.1-eth-0.1, ace-0.1-eth-0.3, ace-0.3-eth-0.1, ace-0.1-eth-1 and ace-1-eth-0.1) and samples of water dilutions without any analyte (pure air). Hence, the total number of classes is 12. The choice of these analytes and concentrations was not affected by any particular application constraint, except that the sensors of selected models show consistent and diverse responses among the gas classes. The number of samples per class among 58 samples is the following:

• eth-0.1: 6;
• eth-0.3: 4;
• eth-1: 5;
• ace-0.1: 6;
• ace-0.3: 6;
• ace-1: 3;
• ace-0.1-eth-0.1: 4;
• ace-0.1-eth-0.3: 5;
• ace-0.3-eth-0.1: 5;
• ace-0.1-eth-1: 3;
• ace-1-eth-0.1: 3;
• air: 8.

The measurements were split into 5 batches (‘batch’ attribute), where each batch contained records approximately for all gas classes given in a random order. All the batches were acquired within a time period of 4 days to minimize the effect of the long-term internal and environmental noise present in the system. The number of samples per batch among 58 samples is the following:

• day-1-morning: 19;
• day-2-afternoon: 10;
• day-2-morning: 10;
• day-3-morning: 11;
• day-4-afternoon: 8.

The measurement protocol was the following: we delivered 10 μL of the corresponding dilution to the vessel using a micropipette. The vessel was connected to the ventilator ‘Source’ outlet. After 3 min of exposition, the source of the gas vapor was removed from the vessel, and the recovery phase started. During the recovery phase, the system was sampling room air for 2 additional minutes to record the decay in the sensors signals. Note that 2 min of recovery phase was not sufficient to recover the sensors baseline and re-establish again the initial conditions in the gas chamber. Hence, although we acquired 2 min of recovery phase, the system was pumping air until the sensors recovered the baseline and the whole gas sample was exhausted from the gas chamber.

## 3 Signal-processing methods

The readout data was the output voltage of the sensors׳ conditioning circuit. The 16 acquired time-dependent voltages were converted to resistances according to the voltage-divider scheme and the corresponding load resistor. Hence, each data point in the array described the resistance of a sensor *R*(*t*) at a certain time of measurement *t*. The resistance values in the data set were normalized by subtracting the baseline value *R*0=*R*(*t*0) at the starting point of the measurement *t*0 and scaling by factor *R*0, (*R*(*t*)−*R*0)/*R*0. Note that such format of the measured raw data allows for comparison of responses among different sensors. The recorded time-series signal for each sensor were acquired at the sampling frequency of 25 Hz during 5 min, resulting in 7500 data points per time-series of a single sensor.

Previously to computing the low-frequency and high-frequency features, the raw data were pre-processed by a set of digital filters. A median filter was used to remove the spikes in the signals. Then we employed two Butterworth filters of 3rd order: a low-pass filter (cut-off frequency 0.01 Hz) and a high-pass filter (pass-frequency 0.07 Hz) to extract the low/high frequency parts from the original signals, respectively. Note that these low/high frequency signals (output of the two Butterworth filters) are not distributed within the data set.

**Fig. 1:**
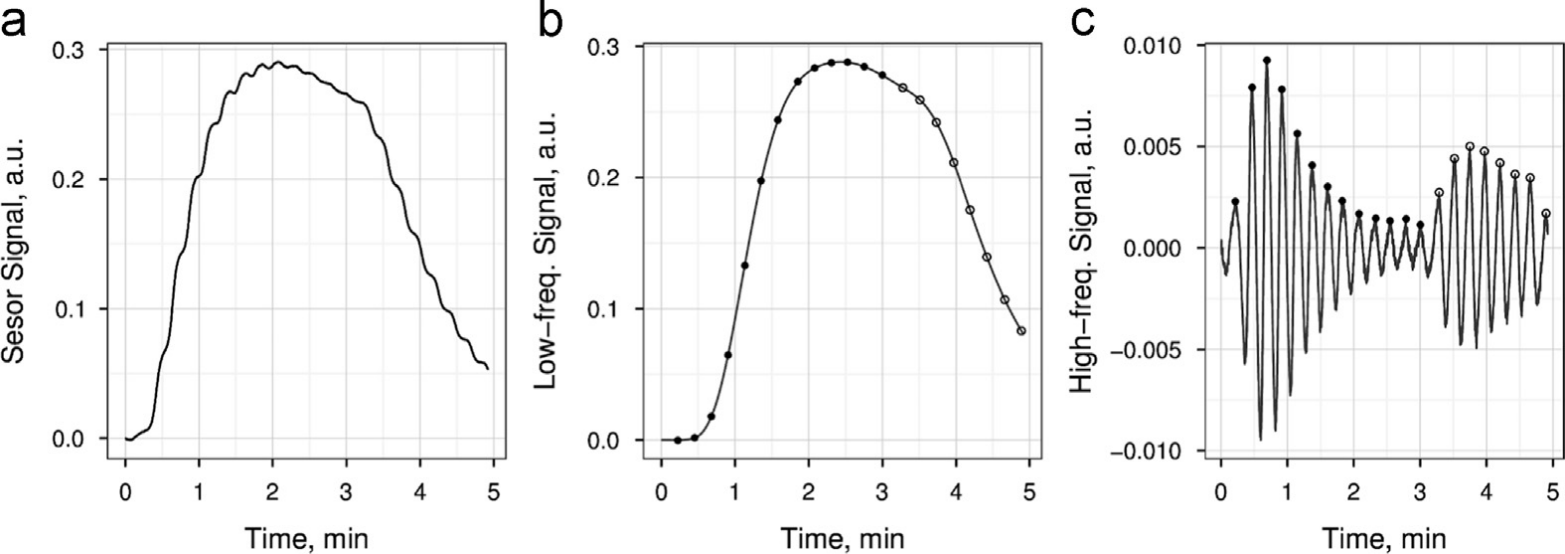
The transient signal recorded from sensor No. 7 in response to sample No. 3 (ethanol at 1 vol.%.) (a). Low-frequency and high-frequency parts of the signals filtered out by the low-pass Butterworth filter with cut-off frequency 0.01 Hz and the high-pass filter with pass-frequency 0.07 Hz, respectively (b) and (c). The solid dots in (b) and (c) panels show 13 features extracted from the transients and stored in the data table of the published data set.

For feature extraction implemented in [1], both low-frequency and high-frequency sensor signals were divided by respiratory cycles, where each cycle was processed independently. Thus, a feature is referred to as a feature by respiratory cycle. Since high-frequency signals showed oscillatory behavior similar to a sine wave curve, we decided to follow a straightforward strategy for feature extraction in this case. We used amplitude of the high-frequency signal (oscillation) at every respiratory cycle as a feature. Low-frequency trajectories had a monotonic behavior, and we used the magnitude of the low-frequency signal as a feature at every respiratory cycle. The value was taken at the same time of oscillation, where the amplitude of the high-frequency signal was measured. Note that the low-frequency and high-frequency features were computed only for the first 13 respiration cycles. Fig. 1 illustrates the feature extraction flow for a single transient of sensor No. 7.

In addition to the low/high frequency features, we also introduced a cycle-independent feature per single measurement, defined as the maximum of the low-frequency signal over the course of the measurement.

## 4 Data sets and attributes

The data published here are organized in two ‘csv’ files, ‘rawdata.csv.gz’ (4.5 MB) and ‘features.csv’ (200 kB). The raw data are stored in the first file ‘rawdata.csv.gz’, where each line represents a single measurement per sensor. Consequently, one needs to read specific 16 consecutive lines to get a single measurement from 16 sensors. The features extracted in [1] are provided in the second file ‘features.csv’, where each line represents features extracted from all 16 time-series of the sensors (a single measurement).

Raw data of each sample contains 16 time-series (one time-series per sensor). Each time-series was recorded during 5 min at a sample rate of 25 Hz (samples per second), providing 7500 data points per time-series. The total number of attributes per sample in raw data is 120,000.

Feature data set includes three types of features extracted from each time-series. Each time-series (one time-series per sensor) is associated with 1 maximum features, 13 high-frequency features and 13 low-frequency features (the features correspond to the first 13 respiration cycles, respectively). The total number of attributes per sample in feature data set is 432.

Both tables of the raw data and features have common attributes:

• ‘exp’: integer (range 100–181); represents the experiment number registered in the laboratory;
• ‘batch’: string (5 values); represents the batch index of the measurements;
• ‘ace_conc’: float (range 0–1); the concentration of the acetone analyte given in vol%;
• ‘eth_conc’: float (range 0–1); the concentration of the ethanol analyte given in vol%;
• ‘lab’: string (12 values); the class label of the gas;
• ‘gas’: string (4 values); another class label that encodes either pure analytes, mixture or air;
• ‘col’: string (12 values); the color code for class labels.

The table of the raw data has specific attributes:

• ‘sensor’: integer (range 1–16); the sensor number;
• ‘sample’: integer (range 1–58); the sample number;
• ‘dR_t〈m〉’: float; represents the value of the time series for a given sensor and for a given sample, which were measured at the time instant 〈m〉, where 〈m〉 takes the value from 1 to 7500.

The table of the features has specific attributes:

• ‘S〈j〉_max’: float; represents the value of the maximum feature extracted from the time-series of sensor 〈j〉;
• ‘S〈j〉_r〈k〉_Alf’: float; represents the low-frequency feature extracted from the time-series of sensor 〈j〉 at the respiration 〈k〉, where 〈j〉 takes the value from 1 to 16, and 〈k〉 takes the value from 1 to 13;
• ‘S〈j〉_r〈k〉_Ahf’: float; represents the high-frequency feature extracted from the time-series of sensor 〈j〉 at the respiration 〈k〉, where 〈j〉 takes the value from 1 to 16, and 〈k〉 takes the value from 1 to 13.

## Figures and Tables

**Table 1:** Configurations of 16 metal-oxide sensors based on the Figaro model [2], the heater voltage and the load resistor. The sensor indices given in the first column correspond to those values reported in the data tables of the published data set.

**Sensor index**	**Sensor model**	**Heater voltage (V)**	**Load resistor (kΩ)**
1	TGS 2610	5.0	21
2	TGS 2610	5.0	21
3	TGS 2602	5.0	21
4	TGS 2600	5.0	21
5	TGS 2610	5.0	21
6	TGS 2611	5.0	21
7	TGS 2610	5.0	21
8	TGS 2620	5.0	21
9	TGS 2610	3.3	82
10	TGS 2620	3.3	82
11	TGS 2602	3.3	82
12	TGS 2611	3.3	82
13	TGS 2610	3.3	82
14	TGS 2610	3.3	82
15	TGS 2610	3.3	82
16	TGS 2600	3.3	82
